# Impact of professional identity on learning engagement among English majors: the chain mediating role of core self-evaluation and critical thinking

**DOI:** 10.3389/fpsyg.2026.1787297

**Published:** 2026-05-11

**Authors:** Bihan Lei, Yuhua Deng, Ruisi Zhang, Na Zhang

**Affiliations:** 1 School of Languages and Cultures, Hunan Institute of Technology, Hengyang, Hunan, China; 2 School of Languages, Literacies and Translation, Universiti Sains Malaysia, Penang, Malaysia; 3 Department of Epidemiology and Health Statistics, Hunan Provincial Key Laboratory of Clinical Epidemiology, Xiangya School of Public Health, Central South University, Changsha, Hunan, China

**Keywords:** English major, college students, professional identity, academic engagement, core self-evaluations, critical thinking

## Abstract

**Background:**

In the context of rapid advancements in intelligent language technology, issues concerning professional identity and learning engagement among English majors have become increasingly prominent. Some students experience unclear professional identity and insufficient academic engagement, which affects the quality of talent cultivation.

**Objective:**

This study explored the influence of professional identity on academic engagement among English majors by examining the sequential mediating effects of core self-evaluation and critical thinking.

**Methods:**

A total of 327 English major undergraduates were recruited via cluster random sampling between March and April 2025. They completed validated scales measuring professional identity, academic engagement, core self-evaluation, and critical thinking. We conducted Pearson’s correlation analysis to examine the bivariate correlations among the study variables. We applied the bias-corrected nonparametric percentile bootstrap method to test the mediating effects.

**Results:**

Significant positive correlations were observed between professional identity, academic engagement, core self-evaluation, and critical thinking among English major undergraduates (*p* < 0.05). The mediating effect test showed that the direct predictive effect of professional identity on academic engagement among English major undergraduates was significant (*p* < 0.05). Both the simple mediating and serial mediating roles of core self-evaluation and critical thinking in the relationship between professional identity and academic engagement were significant. Specifically, the value of the serial mediating effect was 0.021, 95% CI [0.003, 0.044], accounting for 3.13% of the total effect.

**Conclusion:**

The professional identity of English majors can directly predict academic engagement. It also exerts an indirect influence on academic engagement through simple mediators such as core self-evaluation and critical thinking, as well as through chain mediation involving both.

## 1 Introduction

The English major program serves as a vital platform for cultivating talents in cross-cultural communication. As various language tools and digital technologies continue to evolve, the knowledge structure and competency expectations for English professionals are also transforming, presenting new challenges to current educational models. Survey data suggest that in recent years, both the career alignment rate and job satisfaction among English major graduates have exhibited a fluctuating decline (Al-Mahrooqi and Denman, 2016). In this context, stimulating and sustaining English majors’ intrinsic motivation to learn has emerged as a key factor in ensuring the quality of talent cultivation.

Academic engagement, as a positive mental state and emotional disposition, reflects the vitality, dedication, and focus that students demonstrate during the learning process. It serves as a crucial indicator for measuring college students’ growth experiences and the quality of higher education (Chen et al., 2023) and is closely linked to students’ academic achievements and the attainment of professional talent development objectives (Namaziandost et al., 2024). Professional identity, as an integrated psychological state encompassing an individual’s recognition of the value of their major, emotional acceptance, and behavioral commitment, serves as a vital link connecting perceptions of the external environment with internal learning behaviors (Amin et al., 2026; Matsuyama et al., 2021). Existing research indicates that students’ professional identity is a key pathway to enhancing academic engagement, exhibiting a significant positive predictive effect on academic engagement (Liao et al., 2024; Zou et al., 2024). Therefore, when college students develop a high level of recognition of their chosen major, it encourages them to actively engage in their studies and facilitates positive academic development (Young and Dawes, 2024; King et al., 2024).

Although a large body of research has examined the relationship between learning engagement and professional identity among college students (Liao et al., 2024; Zou et al., 2024), studies that specifically focus on English major undergraduates remain relatively scarce. Furthermore, how professional identity affects learning engagement among English majors and whether more complex psychological pathways underlie this relationship remain to be investigated. Early research has indicated that academic engagement is primarily shaped by personality traits (Tisu et al., 2020). Core self-evaluation is a trait-like characteristic that reflects individuals’ fundamental assessments of their own ability and self-worth (Akosile and Ekemen, 2022). Existing studies have reported a reciprocal predictive relationship between core self-evaluation and professional identity, such that students with lower professional identity typically exhibit lower levels of core self-evaluation (Wang et al., 2023). Meanwhile, core self-evaluation has also been confirmed to be significantly and positively correlated with academic engagement (Wang et al., 2023; Paloș et al., 2023). Core self-evaluation consists of four components: self-esteem (one’s overall evaluation of self-worth), general self-efficacy (one’s overall belief in their ability to accomplish tasks), locus of control (whether individuals attribute outcomes to themselves or external factors), and emotional stability (the extent to which one’s emotional state is steady and less prone to anxiety) (Paloș et al., 2023). Research has shown that core dimensions of core self-evaluation often play a partial mediating role in the associations between academic engagement/professional identity and other variables (e.g., mindfulness, peer context) (Chen et al., 2025; Woreta et al., 2025). However, limited research has examined the mediating effect of core self-evaluation in the relationship between professional identity and academic engagement. Therefore, this study aims to investigate the mediating effect of core self-evaluation between professional identity and academic engagement among English-major undergraduates.

As one of the core competencies for the 21st century, critical thinking has become an important developmental goal in higher education worldwide. Driven by rapid technological advancement and economic globalization, society has placed higher demands on university students, who are increasingly expected to possess critical thinking to adapt to complex and changing environments as well as stringent career requirements. As a higher-order rational thinking ability, critical thinking not only constitutes a key component of English majors’ core competencies such as cross-cultural communication and textual analysis, but also serves as essential support for individuals to make rational decisions and achieve self-improvement (Lv et al., 2022; Halpern, 2013). Studies have found that critical thinking is a key antecedent variable influencing students’ learning processes. It not only directly and significantly predicts academic engagement, but also relates to engagement through learning strategies, goal orientation, and academic self-efficacy (Lv et al., 2022; Stavropoulou et al., 2025). Research has suggested that core self-evaluation, as a stable positive self-perception, can provide an important psychological foundation for critical thinking. Individuals with higher core self-evaluation tend to be more confident and internalized, and are more inclined to engage in in-depth thinking, rational analysis and reflective processing, thereby supporting the development and application of critical thinking (Yildiz, 2022; Cristofaro et al., 2020). Thus, core self-evaluation and critical thinking may form a sequential cognitive chain. In addition, researchers have noted that critical thinking is closely associated with professional identity (Ding et al., 2022). Previous studies have shown that critical thinking mediates the relationship between problem-solving ability and academic engagement, and forms a serial mediating pathway with autonomous learning ability (Huang et al., 2023). Another path model study among college students also found that critical thinking mediates the relationship between self-efficacy and self-regulation, thereby relating to academic achievement (Stavropoulou et al., 2025). However, direct empirical evidence remains limited regarding whether critical thinking serves as a mediator between professional identity and academic engagement, and whether it can form a serial mediating pathway with core self-evaluation. Therefore, this study aims to examine the mediating effect of critical thinking between professional identity and academic engagement, as well as their serial mediating mechanism with core self-evaluation among English-major undergraduates.

To clarify the above mechanisms from a systematic theoretical perspective, this study integrates the Situated Demand-Resource (SD-R) model and Control-Value Theory (CVT) to construct the research framework. According to the SD-R model, learning resources can facilitate academic engagement (Lesener et al., 2020). Learning resources include personal resources and environmental resources. In this study, professional identity can be regarded as a combination of personal and environmental resources, whereas core self-evaluation and critical thinking represent typical personal resources. Together, these resources form the psychological foundation for English majors to cope with academic challenges and sustain engagement. As Hobfoll (2011) noted, individuals not only use resources to address difficulties but also accumulate resources for future challenges. Resources do not exist in isolation; instead, they are interrelated, mutually supportive, and synergistically enhancing. Thus, available resources influence individuals’ ability to obtain new resources (Robayo-Tamayo et al., 2020); the more resources one possesses, the more new resources can be continuously acquired, further supporting goal pursuit and activity engagement (Ma et al., 2022). According to CVT, academic emotions depend on individuals’ perceived control and value of learning tasks (Lazarides and Raufelder, 2021; Pekrun, 2006). As a cognitive appraisal pattern reflecting value and control (Tomlinson and Jackson, 2021; Cooper et al., 2017), professional identity can elicit positive academic emotions (Pekrun and Stephens, 2010), which in turn relate to higher academic engagement (Bu, 2024). Integrating the resource accumulation logic of the SD-R model, professional identity may act as an initial personal resource and, through a resource gain pathway, promote the development and accumulation of core self-evaluation (a basic psychological resource) and critical thinking (a higher-order cognitive resource), thereby jointly enhancing academic engagement. Furthermore, core self-evaluation and critical thinking strengthen individuals’ perceived control and value, and through the cognitive–affective transformation mechanism of CVT, ultimately relate to improved academic engagement.

Based on the aforementioned theories and findings, we propose the following hypotheses: (1) H1: Professional identity of English majors exerts a significant positive predictive effect on their academic engagement; (2) H2: Core self-evaluations play a mediating role between professional identity and academic engagement among English-major undergraduates; (3) H3: Critical thinking plays a mediating role between professional identity and academic engagement among English-major undergraduates; and (4) H4: Core self-evaluations and critical thinking exert a serial mediating role between professional identity and academic engagement among English-major undergraduates.

In summary, this study focuses on English major undergraduates and constructs a theoretical model with core self-evaluation and critical thinking as chain mediators, systematically exploring the psychological mechanisms and cognitive pathways through which professional identity influences learning engagement. The findings provide empirical evidence and practical implications for optimizing the cultivation of English professionals.

## 2 Subjects and methods

### 2.1 Research participants

Data were collected between March and April 2025. Using cluster random sampling, the final valid sample comprised 327 undergraduate students majoring in English. Inclusion criteria: ① full-time undergraduate students majoring in English (including English language and literature, translation, business English, and other clearly defined English-related specializations); ② possessing normal cognitive comprehension and language expression abilities and capable of independently completing the questionnaire; and ③ voluntary participation after fully understanding the study’s purpose and content. Exclusion criteria: ① non-English majors and non-undergraduate English students (e.g., graduate students, junior college students); ② students with severe physical or mental health conditions; ③ questionnaires with any missing responses or those exhibiting obvious response patterns (e.g., straight-lining, alternating extremes); and ④ students who self-reported experiencing major life events (e.g., bereavement, serious illness, parental divorce) within the past 3 months that significantly impaired their ability to concentrate on academic work.

To ensure strict implementation of the above criteria, three researchers (Bihan Lei, Yuhua Deng, and Ruisi Zhang) formed a screening team: (i) participants were confirmed as full-time English majors based on the major and grade information provided in the questionnaire; (ii) a clear explanation of the research purpose, completion requirements, and the anonymity principle was provided on the first page of the questionnaire. This was combined with verbal instructions given offline to ensure that participants provided informed consent voluntarily before proceeding to formal responses; (iii) subjective criteria, such as physical and mental health status and recent major life changes, were assessed through self-report measures, with relevant items placed on the first page of the questionnaire; (iv) after data collection, two researchers (Bihan Lei and Yuhua Deng) independently checked the questionnaires for completeness and logical consistency and excluded those that did not meet the criteria.

The sample size of was determined through priori power analysis. First, G*Power 3.1.9.6 software was used for calculation with a linear multiple regression model (six predicted variables expected), a medium effect size (*f*^2^ = 0.15), *α* = 0.05, and 95% statistical power, yielding a required minimum sample size of 176, which ensures the statistical power of the overall regression model. Second, given that the bootstrap method, which generally requires a larger sample size to detect indirect effects, was employed to assess the significance of the mediating pathways, we conducted a further priori power analysis using MedPower software. Based on an expected medium effect size for the path coefficients, the analysis indicated that a minimum sample of 241 participants was sufficient to achieve 80% statistical power at *α* = 0.05. To ensure adequate power for testing this complex mediation model, a total of 327 valid questionnaires were ultimately collected. This final sample size meets and substantially exceeds the highest requirement derived from the two power analyses, thereby ensuring the robustness of the subsequent statistical inferences.

### 2.2 Research instruments

This study employed the following instruments to collect relevant data: a general information questionnaire, the Chinese version of the Professional Identity Questionnaire for Undergraduate Students (PIQUS) (Wang et al., 2025), the Utrecht Work Engagement Scale–Student (UWES-S) (Wang et al., 2025), the Core Self-Evaluations Scale (CSES) (Judge et al., 2003), and the Critical Thinking Assessment Scale (Searing and Kooken, 2016).

(1) General information survey form: Designed by the researchers, it included details such as gender, grade level, family residence, major selection, and household economic status.
(2) Chinese version of the PIQUS: This questionnaire was used to assess the professional identity of the research participants. It included four dimensions–cognitive identity, emotional identity, behavioral identity, and adaptation identity–with a total of 23 items. All items were scored on a 5-point Likert scale, ranging from 1 (“Strongly Disagree”) to 5 (“Strongly Agree”). The total score ranges from 23 to 115, with higher scores indicating a higher level of professional identity (Wang et al., 2025). This questionnaire has good reliability and validity and has been widely used in studies on the professional identity of Chinese students (Zou et al., 2024); the Cronbach’s *α* coefficient of this questionnaire in the present study was 0.970.
(3) UWES-S: This scale was used to assess the academic engagement of the research participants. It includes three dimensions–vigor, dedication, and absorption–with a total of 17 items. All items are scored on a 7-point Likert scale, ranging from 1 (“Never”) to 7 (“Always/Every day”). The total score ranges from 17 to 119, with higher scores indicating a higher level of academic engagement (Wang et al., 2025). In the present study, Cronbach’s *α* coefficient for this scale was 0.974.
(4) CSES: This scale was used to assess the core self-evaluation level of the research participants. It is a unidimensional scale consisting of 10 items. All items are scored on a 5-point Likert scale, ranging from 1 (“Strongly Disagree”) to 5 (“Strongly Agree”). The total score ranges from 10 to 50, with higher scores indicating a higher level of core self-evaluation among the participants (Judge et al., 2003). In the present study, Cronbach’s *α* coefficient for this scale was 0.915.
(5) Critical thinking assessment scale: This scale was revised based on the California Critical Thinking Dispositions Inventory (CCTDI) (Searing and Kooken, 2016) and the Chinese version of the CCTDI (CTDI-CV) developed by Lv et al. (2022). It also incorporates actual teaching scenarios and suggestions from relevant experts. The scale consists of seven dimensions, including truth-seeking, open-mindedness, analytical ability, systematic ability, confidence in critical thinking, cognitive maturity, and inquisitiveness. Each dimension contains 10 items, totaling 70 items. All items are scored on a 6-point Likert scale, ranging from 1 (“Strongly Disagree”) to 6 (“Strongly Agree”). The total score ranges from 70 to 420, with higher total scores indicating stronger critical thinking abilities. In the present study, Cronbach’s *α* coefficient of this scale was 0.896.

### 2.3 Data collection

This study employed a cluster random sampling method with classes as the sampling units. A total of 12 classes were selected as the study participants, with three classes randomly drawn from each of the four grades (freshman to senior) majoring in English. With the assistance of counselors and class teachers, the researchers organized the survey in person on a class-by-class basis, where students participated voluntarily and anonymously. The questionnaire was distributed online via the “Wenjuanxing” platform.

To ensure data quality, the following quality control measures were implemented during data collection and post-processing: prior to questionnaire completion, the researchers utilized standardized instructions to explain the research purpose and completion requirements to the participants; all questionnaire items were set as mandatory on the platform, thereby technically preventing missing data; and after data collection, questionnaires with excessively short response times or obviously regularized answers (e.g., all options the same, wavy-patterned responses) were excluded through manual screening and the platform’s logical verification function.

Based on this process, a total of 435 students were invited to participate in the survey, and 370 electronic questionnaires were returned, yielding a response rate of 85.06%. After strict screening, 327 valid questionnaires were obtained, with an effective response rate of 88.38%.

### 2.4 Statistical analysis

SPSS version 23.0 was used for statistical analysis. Measurement data that conformed to a normal distribution are expressed as mean ± standard deviation (x̄ ± SD). Independent samples *t*-tests or one-way analysis of variance (ANOVA) were used for comparisons between groups. Pearson’s correlation analysis was used to examine the relationships among variables.

Before conducting the mediating effect analysis, Harman’s single-factor test was performed to assess common method bias, and multicollinearity analysis was conducted among the variables. Model 6 of the PROCESS (version 3.5) plugin was used for model testing. The bias-corrected nonparametric percentile bootstrap method (with a bootstrap sample size of 5,000) was adopted to correct the standard error. Bias-corrected confidence intervals were used to determine the significance of mediating effects: a mediating effect was indicated if the 95% confidence interval (CI) did not include 0. *p* < 0.05 was considered statistically significant.

## 3 Results

### 3.1 Scale scores

This study included 327 English majors with the following scale scores: the PIQUS scale yielded a total score of (86.65 ± 5.20), the UWES-S scale scored (74.54 ± 10.07), the Critical Thinking Assessment Scale recorded (275.59 ± 18.05), and the CSES scale achieved (36.47 ± 3.78). Detailed data are presented in Table 1.

**Table 1:** Scale scores ( $\bar{x}$ ±SD, points).

Project	Number of entries	Total score	Entry mean
PIQUS scale	23	86.65 ± 5.20	3.77 ± 0.23
Cognitive identity	5	19.18 ± 2.11	3.84 ± 0.42
Emotional identity	8	30.86 ± 3.58	3.86 ± 0.45
Behavioral identity	6	22.46 ± 2.71	3.74 ± 0.45
Adaptation identity	4	14.15 ± 2.42	3.54 ± 0.60
UWES-S scale	17	74.54 ± 10.07	4.39 ± 0.59
Vigor	6	25.15 ± 6.08	4.19 ± 1.01
Dedication	5	23.21 ± 5.76	4.64 ± 1.15
Absorption	6	26.18 ± 6.15	4.36 ± 1.02
Critical thinking assessment scale	70	275.59 ± 18.05	3.94 ± 0.26
Truth-seeking	10	37.47 ± 6.35	3.75 ± 0.64
Open-mindedness	10	39.59 ± 5.98	3.96 ± 0.60
Analytical ability	10	41.28 ± 7.42	4.13 ± 0.74
Systematic ability	10	37.49 ± 5.35	3.75 ± 0.53
Self confidence	10	38.26 ± 7.97	3.83 ± 0.80
Cognitive maturity	10	40.21 ± 8.37	4.02 ± 0.84
Inquisitiveness	10	41.29 ± 7.15	4.13 ± 0.72
CSES scale	10	36.47 ± 3.78	3.65 ± 0.38

### 3.2 Academic engagement among research participants with different demographic characteristics

The findings reveal statistically significant differences (*p* < 0.05) in academic engagement assessment scores among English majors across different grade levels and program selection pathways, as detailed in Table 2. The General Information Survey Form was designed by researchers. It includes details such as gender, grade level, family residence, major selection, and household economic status.

**Table 2:** Comparison of academic engagement among subjects with different demographic characteristics ( $\bar{x}$ ±s, points).

Project	*N*	UWES-S scale score	*F/t* value	*p* value
Gender			1.339	0.182
Male	47	76.36 ± 9.59		
Female	280	74.24 ± 10.13		
Grade level			4.179	0.006
Grade 1	77	75.22 ± 10.34		
Grade 2	73	77.41 ± 10.05		
Grade 3	85	74.21 ± 9.62		
Grade 4	92	72.01 ± 9.75		
Family residence			1.537	0.125
Rural	175	73.75 ± 10.51		
City	152	75.46 ± 9.49		
Major selection			5.837	0.003
Self-directed choice	214	75.66 ± 10.20		
Choices made by parents or others	69	73.90 ± 8.88		
Assigned major	44	70.14 ± 10.11		
Household economic status			1.110	0.268
Financial hardship	95	73.58 ± 9.39		
No-financial hardship	232	74.94 ± 10.33		

### 3.3 Correlation analysis

**Figure 1:**
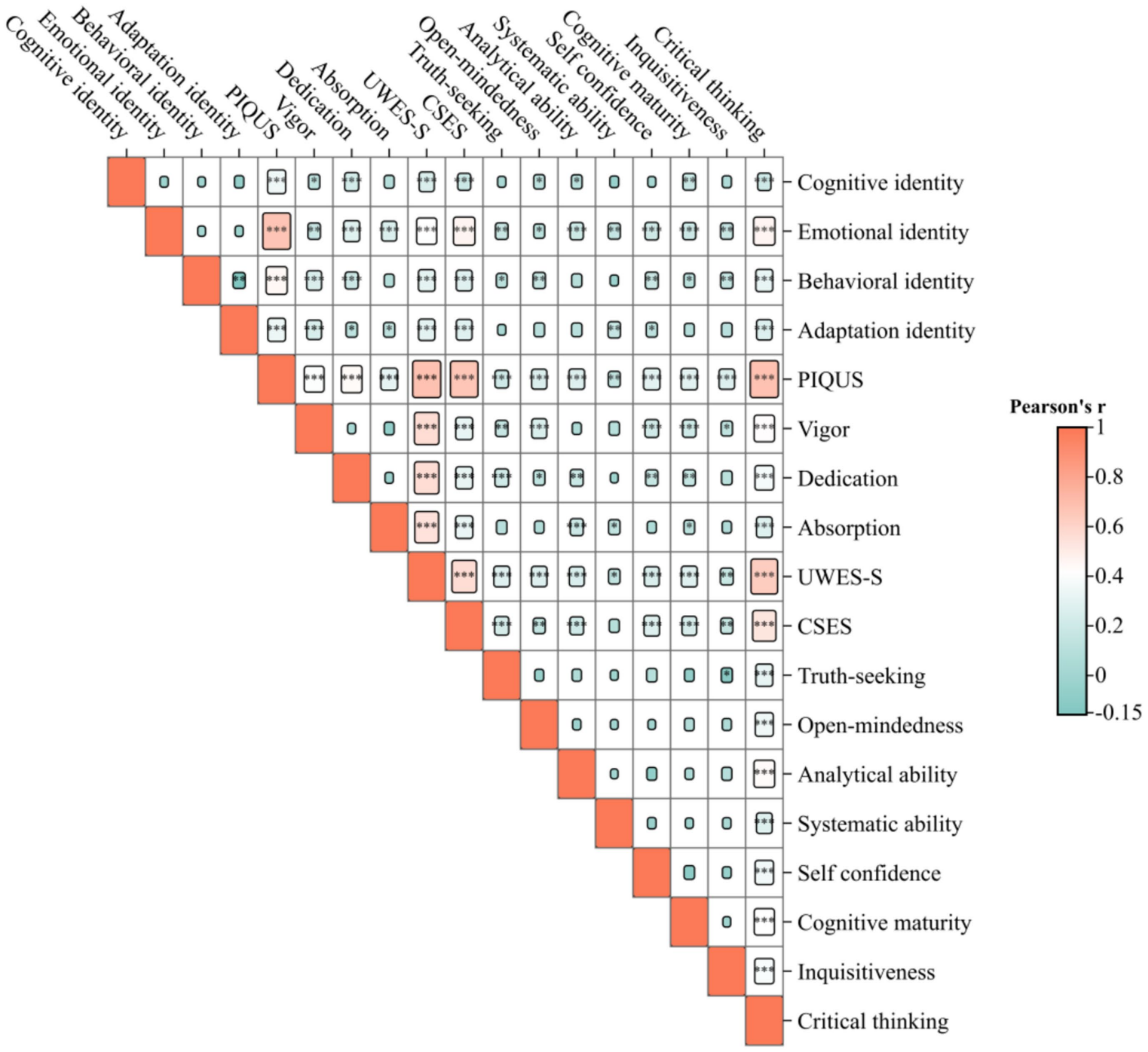
Correlation analysis of professional identity, academic engagement, core self-evaluations, and critical thinking among English-major undergraduates. “*“in the grid indicates *p* < 0.05, “**”indicates *p* < 0.01, and “***“indicates *p* < 0.001; PIQUS (Professional Identity Questionnaire for Undergraduate Students), UWES-S (Utrecht Work Engagement Scale-Student), CSES (Core Self-Evaluations Scale).

The results of the Pearson correlation analysis showed significant positive correlations between each pair of variables, including professional identity, academic engagement, core self-evaluations, and critical thinking (*p* < 0.05). Details are presented in Figure 1 and Appendix 1.

### 3.4 Analysis of mediating effects

Unrotated exploratory factor analysis was conducted for all scale items using Harman’s single-factor test. The results showed that eight factors with eigenvalues greater than 1 were extracted, and the variance explanation rate of the largest common factor was 17.23% (less than 40%), indicating that there was no obvious common method bias in this study. Multicollinearity diagnosis indicated that the variance inflation factor (VIF) values of all variables were lower than the critical value of 5, suggesting that multicollinearity was not a serious problem and would not threaten the interpretation of the results.

**Figure 2:**
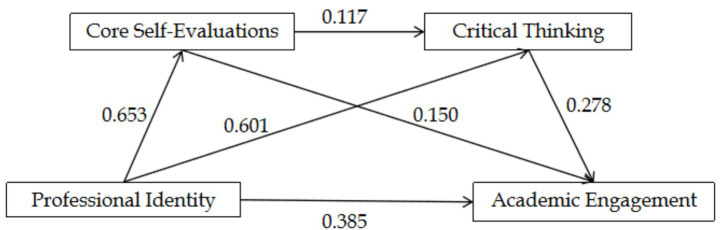
The serial mediation model of core self-evaluations and critical thinking between professional identity and academic engagement.

In this study, a serial mediation model was constructed with academic engagement as the dependent variable, professional identity as the independent variable, and core self-evaluation and critical thinking as mediating variables, including grade level and major selection as control variables (Figure 2). The results showed that after controlling for grade level and major selection, the direct effect of professional identity on academic engagement was significant (*β* = 0.385, *p* < 0.05); core self-evaluation and critical thinking played a partial mediating role between professional identity and academic engagement: professional identity positively predicted core self-evaluation (*β* = 0.653, *p* < 0.05) and critical thinking (*β* = 0.601, *p* < 0.05); core self-evaluation positively predicted critical thinking (*β* = 0.117, *p* < 0.05) and academic engagement (*β* = 0.150, *p* < 0.05); and critical thinking positively predicted academic engagement (*β* = 0.278, *p* < 0.05). Details are presented in Table 3.

**Table 3:** Estimates of standardized path coefficients.

Model	Outcome variable	Predictor variable	*R* value	*R*^2^ value	*F* value	Regression coefficient		
						*Β* value	SE value	*t* value
1	Academic engagement	Professional identity	0.704	0.496	105.782	0.672	0.040	16.864*
		Grade level				−0.053	0.035	−1.514
		Major selection				−0.196	−0.055	−3.573*
2	Core Self-Evaluations	Professional identity	0.680	0.463	92.673	0.653	0.041	15.895*
		Grade level				−0.107	0.036	−2.949*
		Major selection				−0.035	0.057	−0.608
3	Critical Thinking	Professional identity	0.697	0.485	75.932	0.601	0.054	11.168*
		Core Self-Evaluations				0.117	0.055	2.150*
		Grade level				−0.026	0.036	−0.712
		Major selection				−0.095	0.056	−1.699
4	Academic engagement	Professional identity	0.744	0.553	79.483	0.386	0.059	6.520*
		Core Self-Evaluations				0.150	0.051	2.927*
		Critical Thinking				0.278	0.052	5.339*
		Grade level				−0.027	0.034	−0.789
		Major selection				−0.164	0.052	−3.142*

Control variables are coded as follows: grade level (0 = Grade 1, 1 = Grade 2, 2 = Grade 3, 3 = Grade 4); major selection (0 = self-selected, 1 = selected by parents or others, 2 = assigned major); ^*^p < 0.05.

## 4 Discussion

This study investigated the association between professional identity and academic engagement among English-major undergraduates, and further examined the serial mediating effects of core self-evaluation and critical thinking. The results indicate that professional identity not only directly predicts academic engagement but also through three indirect pathways: via the mediating effect of core self-evaluation, via the mediating effect of critical thinking, and via the chained mediating pathway from “core self-evaluation to critical thinking.” Hypotheses 1, 2, 3, and 4 were all supported.

This study finds that there is a significant positive correlation between professional identity and academic engagement among English majors. The results suggest that enhancing professional identity among English majors may contribute to promoting their level of academic engagement. Professional identity, as an integrated state of students’ cognitive, affective, and behavioral dispositions toward their major, essentially reflects their value judgments (recognizing the value of the major and clarifying the meaning of learning) and perceived control (believing in their ability to excel in the major and achieve professional development) regarding academic tasks related to their major (Tomlinson and Jackson, 2021; Cooper et al., 2017). Specifically, English major undergraduates with a high level of professional identity are able to recognize the value of their major—such as cross-cultural communication and language application—at the cognitive level, embrace the learning process and its challenges at the emotional level, and willingly invest time and effort in deepening their professional knowledge and skills at the behavioral level. This integrated psychological state is accompanied by higher perceived value and control appraisal in English learning tasks (Tang, 2020; Pekrun, 2024). According to CVT, when students perceive that a learning task has high value and that they themselves have sufficient control over it (i.e., high professional identity), they are more likely to generate positive academic emotions such as enjoyment and satisfaction (Pekrun and Stephens, 2010). Such positive emotions are directly associated with learners’ professional learning behaviors and sustained academic engagement (Bu, 2024), as well as positively related to self-regulation ability and learning effort, thus connecting to a more stable and enduring learning process (Shao et al., 2019; Appleton et al., 2008). This provides a theoretical explanation for how English majors’ professional identity is positively associated with their level of academic engagement through cognitive appraisal and academic emotions (Table 4).

**Table 4:** Test of the serial mediation effect of core self-evaluations and critical thinking between professional identity and academic engagement.

Path/effect type	Effect value	*SE*	95%CI	Share of total effect
Total effect	0.672	0.040	0.539–0.750	100%
Direct effect	0.386	0.059	0.269–0.502	57.44%
Total indirect effect	0.286	0.049	0.194–0.388	42.56%
Professional identity → core self-evaluations → academic engagement	0.098	0.037	0.028–0.173	14.58%
Professional identity → critical thinking → academic engagement	0.167	0.040	0.093–0.248	24.85%
Professional identity → core self-evaluations → critical thinking → academic engagement	0.021	0.011	0.003–0.044	3.13%

The results of this study indicate that core SEs are positively correlated with both professional identity and academic engagement among English majors, and that core SEs partially mediate the relationship between professional identity and academic engagement. There are two pathways linking professional identity to core self-evaluation: first, by directly generalizing the recognition of the major’s value to positive judgments of one’s own abilities, and second, by fostering positive academic emotions and accumulating and strengthening self-worth and competence beliefs (Brehmer and Strauser, 2023; Karanikola et al., 2018). Moreover, the enhancement of core SEs further promotes English majors’ academic engagement. Studies have shown that students with positive core SEs are better at managing affairs, coping with academic challenges, and proactively engaging in complex tasks and setting higher goals (Gibson and Hicks, 2018). They believe that they can take charge of their academic outcomes through their own abilities and efforts (Pekrun and Stephens, 2010), and the sense of satisfaction derived from academic success further reinforces their self-efficacy, hope, and optimism (Paloș et al., 2023). Therefore, a higher level of core self-evaluation is generally associated with more positive academic cognitive appraisals and more effective mobilization of personal resources (Chang et al., 2012), and may be indirectly linked to learning motivation and engagement through academic-related cognitive and emotional experiences.

Meanwhile, this study also shows that there is a positive correlation between critical thinking and both professional identity and academic engagement among English majors, and that critical thinking plays a partial mediating role between professional identity and academic engagement, which is consistent with the findings of Phan (2009). Based on the above CVT, professional identity reflects English majors’ positive value judgment and perceived control over their learning, which is positively associated with positive academic emotions and thus linked to academic engagement. However, this pathway from cognitive appraisal to emotion to engagement often relies on individuals’ capacity for deep processing of cognitive information. Research indicates that emotions are not directly caused by events but are mediated by individuals’ cognitive beliefs and thinking processes (Turner et al., 2025). As a higher-order cognitive processing ability, critical thinking is precisely the core embodiment of this cognitive mediating link. Additionally, studies indicate that critical thinking is associated with the generation of positive emotions and the regulation of negative emotions (Namaziandost et al., 2023). Taken together, the mediating role of critical thinking between professional identity and academic engagement may be attributed to the following: professional identity provides English majors with cognitive materials regarding professional values, whereas critical thinking deeply processes and transforms these materials. Students with a high level of critical thinking tend to systematically analyze the multidimensional aspects of professional value and translate abstract meanings into personal ones, thus deepening the positive cognitive appraisal associated with professional identity (Ooi, 2022). The positive cognitive appraisal deepened by critical thinking is generally accompanied by a stronger sense of professional value and perceived control, as well as more frequent positive academic emotions such as pleasure and satisfaction, and is ultimately associated with higher levels of academic engagement (Pekrun and Stephens, 2010; Bu, 2024; Shu-Ling and Cherng, 2023).

The results of this study showed that although the effect size of the serial mediating effect of core self-evaluation and critical thinking between professional identity and academic engagement was small, it was still significant. This suggests that English majors with high professional identity can achieve greater academic engagement through the sequential mediating mechanism of strengthening core self-evaluations and promoting critical thinking. The chain-like mediation between core self-evaluation and critical thinking fundamentally reflects the sequential synergistic mechanism between foundational psychological resources and contextual cognitive abilities. Specifically, high levels of professional identity provide the necessary intrinsic motivation and psychological safety foundation for developing discerning, integrative thinking (i.e., critical thinking) tailored to technological application scenarios by reinforcing students’ self-worth affirmation and psychological adaptability amid technological change (i.e., enhancing core self-evaluation) (Judge et al., 2003; Chiang et al., 2014). Building upon this foundation, such technology-context-adapted critical thinking further serves as a crucial cognitive bridge, transforming positive professional identity and a stable self-concept into sustained, deep, and reflective learning behaviors. Furthermore, the finding of this chain mediation path is consistent with the SD-R model (Lesener et al., 2020) and aligns with the gain spiral principle of the “resource caravan” (Hobfoll, 2011; Robayo-Tamayo et al., 2020; Ma et al., 2022). In other words, professional identity, as an initial resource, may form a sequential positive association of “professional identity → core self-evaluation → critical thinking → academic engagement” through the resource gain pathway. The significance of this chained intermediary pathway has practical value. Empirically, it confirms that the professional development of English majors relies not only on direct emotional identification or isolated skill training but, more crucially, on a coherent support process spanning psychological empowerment to cognitive transformation. This provides a precise entry point for educational interventions: while enhancing students’ professional identity, systematic attention should be given to building their overall self-concept. On this foundation, efforts should focus on cultivating their critical literacy in technological contexts, thereby forming an integrated psychological–cognitive foundation that supports deep and sustained academic engagement.

This study has several limitations. First, because of the adoption of a cross-sectional research design, it has certain limitations in verifying causality. In future research, longitudinal follow-up surveys could be conducted to explore the causal relationship between professional identity and academic engagement among English major undergraduates. Second, restricted by research funding and time, this study conducted a questionnaire survey in only one university. Therefore, the generalizability of the research results should be treated with caution. Third, all data in this study were collected through student self-report questionnaires, which may lead to certain subjective biases.

## 5 Conclusion

The professional identity of English majors exerts a significant positive predictive effect on their academic engagement. It can not only directly and positively predict academic engagement but also indirectly predict it through the sequential mediating pathway of “core self-evaluations to critical thinking.” Based on this mechanism, the level of academic engagement among English majors can be effectively enhanced through a comprehensive strategy that improves their professional identity, strengthens their core self-evaluations, and systematically cultivates their critical thinking skills.

## Data Availability

The original contributions presented in the study are included in the article/Supplementary material, further inquiries can be directed to the corresponding author.
