# Supplementary material for: Impact of professional identity on learning engagement among English majors: the chain mediating role of core self-evaluation and critical thinking
Source: Front Psychol. 2026 May 11;17:1787297. doi: 10.3389/fpsyg.2026.1787297 (PMC13199295; doi:10.3389/fpsyg.2026.1787297)
Supplement: Supplementary file 1 [file Supplementary_file_1.docx]

**Appendix 1**：Specific results of correlation analysis

| Project |  | Cognitive identity | Emotional identity | Behavioral identity | Adaptation identity | **PIQUS** | Vigor | Dedication | Absorption | **UWES-S** | **CSES** | Truth-seeking | Open-mindedness | Analytical ability | Systematic ability | Self confidence | Cognitive maturity | Inquisitiveness | **Critical Thinking** |
| --- | --- | --- | --- | --- | --- | --- | --- | --- | --- | --- | --- | --- | --- | --- | --- | --- | --- | --- | --- |
| Cognitive identity | r value | 1.000 | -0.026 | -0.008 | -0.065 | 0.353 | 0.129 | 0.221 | 0.081 | 0.254 | 0.183 | 0.031 | 0.118 | 0.109 | -0.047 | 0.022 | 0.175 | 0.057 | 0.194 |
|  | P value |  | 0.635 | 0.886 | 0.242 | ＜0.001 | 0.019 | ＜0.001 | 0.145 | ＜0.001 | 0.001 | 0.581 | 0.033 | 0.049 | 0.398 | 0.686 | 0.002 | 0.305 | ＜0.001 |
| Emotional identity | r value | -0.026 | 1.000 | 0.013 | -0.023 | 0.674 | 0.167 | 0.283 | 0.255 | 0.419 | 0.465 | 0.171 | 0.116 | 0.191 | 0.157 | 0.198 | 0.185 | 0.164 | 0.463 |
|  | P value | 0.635 |  | 0.814 | 0.673 | ＜0.001 | 0.002 | ＜0.001 | ＜0.001 | ＜0.001 | ＜0.001 | 0.002 | 0.036 | 0.001 | 0.005 | ＜0.001 | 0.001 | 0.003 | ＜0.001 |
| Behavioral identity | r value | -0.008 | 0.013 | 1.000 | -0.158 | 0.452 | 0.246 | 0.185 | 0.079 | 0.303 | 0.281 | 0.113 | 0.163 | 0.092 | -0.029 | 0.179 | 0.110 | 0.160 | 0.317 |
|  | P value | 0.886 | 0.814 |  | 0.004 | ＜0.001 | ＜0.001 | 0.001 | 0.153 | ＜0.001 | ＜0.001 | 0.041 | 0.003 | 0.095 | 0.600 | 0.001 | 0.047 | 0.004 | ＜0.001 |
| Adaptation identity | r value | -0.065 | -0.023 | -0.158 | 1.000 | 0.340 | 0.243 | 0.127 | 0.126 | 0.296 | 0.277 | 0.027 | 0.103 | 0.099 | 0.166 | 0.123 | 0.081 | 0.104 | 0.267 |
|  | P value | 0.242 | 0.673 | 0.004 |  | ＜0.001 | ＜0.001 | 0.021 | 0.023 | ＜0.001 | ＜0.001 | 0.622 | 0.063 | 0.073 | 0.003 | 0.026 | 0.146 | 0.059 | ＜0.001 |
| **PIQUS** | r value | 0.353 | 0.674 | 0.452 | 0.340 | 1.000 | 0.408 | 0.440 | 0.308 | 0.686 | 0.669 | 0.202 | 0.261 | 0.270 | 0.151 | 0.296 | 0.293 | 0.268 | 0.686 |
|  | P value | ＜0.001 | ＜0.001 | ＜0.001 | ＜0.001 |  | ＜0.001 | ＜0.001 | ＜0.001 | ＜0.001 | ＜0.001 | ＜0.001 | ＜0.001 | ＜0.001 | 0.006 | ＜0.001 | ＜0.001 | ＜0.001 | ＜0.001 |
| Vigor | r value | 0.129 | 0.167 | 0.246 | 0.243 | 0.408 | 1.000 | 0.021 | -0.078 | 0.568 | 0.316 | 0.163 | 0.251 | 0.055 | 0.099 | 0.205 | 0.199 | 0.132 | 0.428 |
|  | P value | 0.019 | 0.002 | ＜0.001 | ＜0.001 | ＜0.001 |  | 0.701 | 0.158 | ＜0.001 | ＜0.001 | 0.003 | ＜0.001 | 0.322 | 0.073 | ＜0.001 | ＜0.001 | 0.017 | ＜0.001 |
| Dedication | r value | 0.221 | 0.283 | 0.185 | 0.127 | 0.440 | 0.021 | 1.000 | -0.031 | 0.566 | 0.313 | 0.205 | 0.131 | 0.179 | -0.010 | 0.158 | 0.160 | 0.107 | 0.373 |
|  | P value | ＜0.001 | ＜0.001 | 0.001 | 0.021 | ＜0.001 | 0.701 |  | 0.571 | ＜0.001 | ＜0.001 | ＜0.001 | 0.018 | 0.001 | 0.853 | 0.004 | 0.004 | 0.052 | ＜0.001 |
| Absorption | r value | 0.081 | 0.255 | 0.079 | 0.126 | 0.308 | -0.078 | -0.031 | 1.000 | 0.546 | 0.325 | 0.100 | 0.069 | 0.184 | 0.134 | 0.054 | 0.109 | 0.062 | 0.273 |
|  | P value | 0.145 | ＜0.001 | 0.153 | 0.023 | ＜0.001 | 0.158 | 0.571 |  | ＜0.001 | ＜0.001 | 0.070 | 0.215 | 0.001 | 0.015 | 0.326 | 0.048 | 0.260 | ＜0.001 |
| **UWES-S** | r value | 0.254 | 0.419 | 0.303 | 0.296 | 0.686 | 0.568 | 0.566 | 0.546 | 1.000 | 0.569 | 0.277 | 0.269 | 0.248 | 0.136 | 0.247 | 0.279 | 0.179 | 0.638 |
|  | P value | ＜0.001 | ＜0.001 | ＜0.001 | ＜0.001 | ＜0.001 | ＜0.001 | ＜0.001 | ＜0.001 |  | ＜0.001 | ＜0.001 | ＜0.001 | ＜0.001 | 0.014 | ＜0.001 | ＜0.001 | 0.001 | ＜0.001 |
| CSES | r value | 0.183 | 0.465 | 0.281 | 0.277 | 0.669 | 0.316 | 0.313 | 0.325 | 0.569 | 1.000 | 0.223 | 0.150 | 0.207 | 0.088 | 0.269 | 0.237 | 0.153 | 0.529 |
|  | P value | 0.001 | ＜0.001 | ＜0.001 | ＜0.001 | ＜0.001 | ＜0.001 | ＜0.001 | ＜0.001 | ＜0.001 |  | ＜0.001 | 0.007 | ＜0.001 | 0.111 | ＜0.001 | ＜0.001 | 0.005 | ＜0.001 |
| Truth-seeking | r value | 0.031 | 0.171 | 0.113 | 0.027 | 0.202 | 0.163 | 0.205 | 0.100 | 0.277 | 0.223 | 1.000 | -0.053 | 0.062 | -0.014 | 0.095 | -0.074 | -0.122 | 0.315 |
|  | P value | 0.581 | 0.002 | 0.041 | 0.622 | ＜0.001 | 0.003 | ＜0.001 | 0.070 | ＜0.001 | ＜0.001 |  | 0.337 | 0.266 | 0.805 | 0.085 | 0.181 | 0.027 | ＜0.001 |
| Open-mindedness | r value | 0.118 | 0.116 | 0.163 | 0.103 | 0.261 | 0.251 | 0.131 | 0.069 | 0.269 | 0.150 | -0.053 | 1.000 | -0.031 | -0.008 | -0.008 | 0.073 | 0.057 | 0.351 |
|  | P value | 0.033 | 0.036 | 0.003 | 0.063 | ＜0.001 | ＜0.001 | 0.018 | 0.215 | ＜0.001 | 0.007 | 0.337 |  | 0.580 | 0.889 | 0.880 | 0.187 | 0.305 | ＜0.001 |
| Analytical ability | r value | 0.109 | 0.191 | 0.092 | 0.099 | 0.270 | 0.055 | 0.179 | 0.184 | 0.248 | 0.207 | 0.062 | -0.031 | 1.000 | -0.003 | -0.078 | 0.039 | 0.086 | 0.440 |
|  | P value | 0.049 | 0.001 | 0.095 | 0.073 | ＜0.001 | 0.322 | 0.001 | 0.001 | ＜0.001 | ＜0.001 | 0.266 | 0.580 |  | 0.961 | 0.158 | 0.483 | 0.119 | ＜0.001 |
| Systematic ability | r value | -0.047 | 0.157 | -0.029 | 0.166 | 0.151 | 0.099 | -0.010 | 0.134 | 0.136 | 0.088 | -0.014 | -0.008 | -0.003 | 1.000 | -0.041 | -0.018 | 0.034 | 0.275 |
|  | P value | 0.398 | 0.005 | 0.600 | 0.003 | 0.006 | 0.073 | 0.853 | 0.015 | 0.014 | 0.111 | 0.805 | 0.889 | 0.961 |  | 0.465 | 0.740 | 0.538 | ＜0.001 |
| Self confidence | r value | 0.022 | 0.198 | 0.179 | 0.123 | 0.296 | 0.205 | 0.158 | 0.054 | 0.247 | 0.269 | 0.095 | -0.008 | -0.078 | -0.041 | 1.000 | -0.101 | -0.046 | 0.363 |
|  | P value | 0.686 | ＜0.001 | 0.001 | 0.026 | ＜0.001 | ＜0.001 | 0.004 | 0.326 | ＜0.001 | ＜0.001 | 0.085 | 0.880 | 0.158 | 0.465 |  | 0.067 | 0.410 | ＜0.001 |
| Cognitive maturity | r value | 0.175 | 0.185 | 0.110 | 0.081 | 0.293 | 0.199 | 0.160 | 0.109 | 0.279 | 0.237 | -0.074 | 0.073 | 0.039 | -0.018 | -0.101 | 1.000 | -0.016 | 0.421 |
|  | P value | 0.002 | 0.001 | 0.047 | 0.146 | ＜0.001 | ＜0.001 | 0.004 | 0.048 | ＜0.001 | ＜0.001 | 0.181 | 0.187 | 0.483 | 0.740 | 0.067 |  | 0.780 | ＜0.001 |
| Inquisitiveness | r value | 0.057 | 0.164 | 0.160 | 0.104 | 0.268 | 0.132 | 0.107 | 0.062 | 0.179 | 0.153 | -0.122 | 0.057 | 0.086 | 0.034 | -0.046 | -0.016 | 1.000 | 0.390 |
|  | P value | 0.305 | 0.003 | 0.004 | 0.059 | ＜0.001 | 0.017 | 0.052 | 0.260 | 0.001 | 0.005 | 0.027 | 0.305 | 0.119 | 0.538 | 0.410 | 0.780 |  | ＜0.001 |
| **Critical Thinking** | r value | 0.194 | 0.463 | 0.317 | 0.267 | 0.686 | 0.428 | 0.373 | 0.273 | 0.638 | 0.529 | 0.315 | 0.351 | 0.440 | 0.275 | 0.363 | 0.421 | 0.390 | 1.000 |
|  | P value | ＜0.001 | ＜0.001 | ＜0.001 | ＜0.001 | ＜0.001 | ＜0.001 | ＜0.001 | ＜0.001 | ＜0.001 | ＜0.001 | ＜0.001 | ＜0.001 | ＜0.001 | ＜0.001 | ＜0.001 | ＜0.001 | ＜0.001 |  |

Note: PIQUS :Professional Identity Questionnaire for Undergraduate Students, UWES-S: Utrecht Work Engagement Scale-Student, CSES: Core Self-Evaluations Scale.
